# Multiplex PCR for simultaneous identification of *E. coli* O157:H7, *Salmonella* spp. and *L. monocytogenes* in food

**DOI:** 10.1007/s13205-016-0523-6

**Published:** 2016-09-24

**Authors:** Thuy Trang Nguyen, Vo Van Giau, Tuong Kha Vo

**Affiliations:** 1 Department of Pharmacy, Ho Chi Minh City University of Technology (HUTECH), 475A Dien Bien Phu Street, Ward 25, Binh Thanh District, Ho Chi Minh City, Vietnam; 2 Department of Faculty of Food Technology, Ho Chi Minh City University of Food Industry (HUFI), 140 Le Trong Tan, Tan Phu district, Ho Chi Minh City, Vietnam; 3 Vietnam Sports Hospital, Ministry of Culture, Sports and Tourism, Do Xuan Hop Road, My Dinh I Ward, Nam Tu Liem District, Hanoi, Vietnam; 4 Department of BionanoTechnology, Gachon Medical Research Institute, Gachon University, Sungnam, Korea

**Keywords:** Multiplex PCR, Simultaneous, Detection, Food-borne pathogen

## Abstract

The rapid detection of pathogens in food is becoming increasingly critical for ensuring the safety of consumers, since the majority of food-borne illnesses and deaths are caused by pathogenic bacteria. Hence, rapid, sensitive, inexpensive and convenient approaches to detect food-borne pathogenic bacteria is essential in controlling food safety. In this study, a multiplex PCR assay for the rapid and simultaneous detection of *Escherichia coli* O157:H7, *Salmonella* spp. and *Listeria monocytogenes* was established. The *invA, stx* and *hlyA* genes specifically amplified DNA fragments of 284, 404 and 510 bp from *Salmonella* spp., *L. monocytogenes* and *E. coli* O157:H7, respectively. The 16S rRNA gene was targeted as an internal control gene in the presence of bacterial DNA. The specificity and sensitivity of the multiplex PCR were performed by testing different strains. The multiplex PCR assay was able to specifically simultaneously detect ten colony-forming unit/mL of each pathogen in artificially inoculated samples after enrichment for 12 h. The whole process took less than 24 h to complete, indicating that the assay is suitable for reliable and rapid identification of these three food-borne pathogens, which could be suitable in microbial epidemiology investigation.

## Introduction

Food-borne diseases, which are mainly caused by food-borne pathogens, are a serious health hazard in both developing and developed countries. *Escherichia coli* O157:H7, *Salmonella* spp. and *Listeria monocytogenes* are commonly considered as food-borne pathogens in many countries (Cetinkaya et al. 2014; Modzelewska-Kapituła et al. 2014; Nowak et al. 2007; Wang et al.^ 2007^; Zhou et al. 2014). *Salmonella* illnesses are commonly associated with poultry and eggs, along with meat, unpasteurized milk or juice, cheese, contaminated raw fruits and vegetables, spices and nuts, through consumption of contaminated salmonellosis (Modzelewska-Kapituła et al. 2014; Nowak et al. 2007; Zhou et al. 2014). The *inv*A, a gene of *Salmonella*, contains those sequences that are unique to this genus and has been proved as a suitable PCR target with potential diagnostic applications (Jamshidi et al. 2009). In addition, approximately 634 people were infected by *Salmonella* heidelberg from the farms chicken, according to the recent reports by the Center for Disease Control and Prevention in 2013 and 2014. Listeriosis, a serious infection usually caused by eating food contaminated with the bacterium *L. monocytogenes* (Ahmed et al. 2015), has a high case fatality rate (20–30 %) in immunocompromised populations (Chen and Knabel 2007). It is considered a hazardous agent in the food industry, and *L. monocytogenes* can be detected in a large variety of vegetable food products as well as animal products (Mead et al. 1999). *L. monocytogenes* is found in a variety of foods such as milk, milk products, eggs, poultry and meat (Amagliani et al. 2004). *L. monocytogenes* became a frequent pathogen involved more frequently in sporadic severe illnesses and outbreaks of food-borne infections. *hly*A is the gene that codes for the listeriolysin O toxin which is present in the genome of pathogenic *L. monocytogenes*. Since this gene codes for the toxin of the pathogen, it is necessary for virulence and is used for identifying *L. monocytogenes* in the presence of other *Listeria* strains (Churchill et al. 2006). According to recent reports by the CDC, there were 11 multistate outbreaks of Shiga toxin-producing *E. coli* in the USA from 2011 to 2014 with six of them attributed to *E. coli* O157:H7. The transmission of *E. coli* O157:H7 remains a major public health concern worldwide due to its low infectious dose. It is highly virulent; an inoculation of less than 10–100 CFU of *E. coli* O157:H7 is sufficient to cause infection (Coffey et al. 2011). *E. coli* O157:H7 may cause sporadic or epidemic cases of diarrhea, often with bloody stools, due mainly to the ingestion of contaminated food products (Chase-Topping et al. 2007). Shiga toxin (Stx) is one of the major virulence factors causing hemorrhagic colitis and hemolytic uremic syndrome involved in *E. coli* O157:H7 pathogenesis (Melton-Celsa et al. 2012) and are identified as stx1, stx1c, stxfc, stx2, stx2e, stx2d and stx2g (Gobius et al. 2003). These genes code for the toxin of the pathogen; it is necessary for virulence and useful for identifying *E. coli* O157:H7 in the presence of other strains. Some studies have employed one primer which could detect both the *Stx1* and *Stx2* genes (Aranda et al. 2007; Toma et al. 2003). Hence, the presence of bacterial pathogens in food poses a potential risk of infection in human health. Thus, the development of a rapid, cheap and sensitive method for the detection of these pathogens in food is required not only for improving food safety, but also for protecting human health.

Currently, the proposed approaches for the detection of food-borne pathogens is mainly the culture-based bacterial isolation and identification, but this is probably tedious and time-consuming (2–3 days) (Zhao et al. 2014). The application of genetic-based techniques is able to detect the pathogenic bacteria with greater sensitivity and reliability than conventional culture methods. PCR is more than a promising method; it has been applied with success in various protocols used for pathogen detection (Giau et al. 2016; Lee et al. 2014; Rahn et al. 1992; Zhang et al. 2009), particularly multiplex PCR assay (Xu et al. 2012). Interestingly, multiplex PCR (mPCR) allows multiple gene analysis of bacteria at the same time in a single reaction tube simultaneously, saving time and reagents. A rapid, sensitive and specific method that would allow detection of multiple pathogens simultaneously from different types of foods would be very valuable for the food industry and regulatory agencies. Thus, keeping the above in view, we developed a multiplex PCR for the rapid and simultaneous detection of three epidemic food-borne pathogens: *E. coli* O157:H7, *Salmonella* spp., and *L. monocytogenes.* The performance of the multiplex assay, including its sensitivity, specificity and precision in qualitative analyses, was comprehensively evaluated in comparison with single target assays. The capacity of the proposed assay to detect multiple target pathogens simultaneously was also performed, and the effect of non-target interference on the assay performance was evaluated. The results obtained with artificially contaminated food samples show that the multiplex assay can simultaneously detect these three target food-borne pathogens in foods with high sensitivity and reliability.

## Materials and methods

### Bacterial strains and cultivation conditions

A total of 16 bacterial strains were used for evaluating the specificity of the multiplex PCR assay in this study, including 5 target bacterial strains and 11 other bacterial strains. In which, three strains *E. coli* O157:H7 were obtained from previously worked (Giau et al. 2016). The strains were verified by biochemical and immunologic methods, stored at −80 °C in 10 % glycerol and grown in Luria–Bertani (LB) (Oxoid, UK) at 37 °C as listed in Table 1. The plate count method was used to measure the level of colony-forming unit (CFU) of each bacterium on LB agar medium. In this study, artificially contaminated samples were inoculated using the simultaneous enrichment broth (SEB) for simultaneous enrichment of three pathogenic bacteria, which was described previously by Kobayashi et al. (2009).

**Table 1:** Bacterial strains and their sources employed in this study

No	Bacteria	Serovar	Source
The target strains			
1	*E. coli*	O157:H7	NIHE
2	*E. coli*	O157:H7	HCMUS
3	*E. coli*	O157:H7	NLU
4	*Salmonella enteritidis*		ATCC13076
5	*Listeria monocytogenes*		ATCC19111
The non-target strains			
1	*E. coli*		ATCC 11775
2	*E. coli*		ATCC 25922
3	*E. coli* (Ahmed et al. 2015)		Clinical isolate
4	*E. coli* (Amagliani et al. 2004)		Clinical isolate
5	*E. coli* (Aranda et al. 2007)		Chicken isolate
6	*E. coli* (Cetinkaya et al. 2014)		Beef isolate
7	*E. coli* (Coffey et al. 2011)		Salad isolate
8	*Clostridium perfringens*		ATCC13124
9	*Bacillus cereus*		ATCC11778
10	*Shigella sonnei*		ATCC 9290
11	*V. cholerae*		ATCC17802

*ATCC A*merican Type Culture Collection, *NIHE* National Institute of Hygiene and Epidemiology*, HCMUS* HCM University of Science, *NLU* Nong Lam University

### Pathogen detection by the conventional culture method

For detection of *E. coli* O157:H7, 25 g of each sample was diluted in 225 mL of modified tryptone soya broth (Mtsb, Oxoid, UK), added with novobiocin, homogenized for 2 min at 260 rpm using a Stomacher (Model 400 circulator, Seward, Norfolk, England) and incubated for 18–24 h at 41.5 °C, as well as the remaining steps, according to the ISO 16654 (2001) method. After the enrichment and immunomagnetic concentration steps, the selective and differential isolation of enterohemorrhagic *E. coli* O157:H7 was carried out on MacConkey agar with sorbitol, cefixime, and tellurite (CT-SMAC, Oxoid, UK) and incubated overnight at 42 °C. From each sample, one well-isolated suspected colony was transferred to tryptone soy agar (Oxoid) and incubated for 24 h at 37 °C. Subsequently, one isolate from the subculture was further tested for agglutination with an *E. coli* O157:H7 latex test kit (Becton-Dickinson, USA) for serogroup O157:H7 confirmation. For detection of *Salmonella* spp., each 25 g food sample was diluted with 225 mL of sterile Buffered Peptone Water (Merck, Germany) and pummeled in a Stomacher apparatus for 2 min; the mixture was then incubated for 18 h at 37 °C. One milliliter of the culture was added to 10 mL of Rappaport–Vassiliadis soy peptone broth (Merck, Germany) and incubated at 42.5 °C for 18 h. One loopful of the culture was then streaked onto xylose lysine deoxycholate agar (Merck, Germany) and incubated at 37 °C for 24 h. The resulting presumptive *Salmonella* colonies were tested with biochemical screening and serological confirmation using *Salmonella* polyvalent O, O1 antisera (Becton–Dickinson, USA). For detection of *L. monocytogenes*, 25 g of the food samples was mixed with 225 mL of sterile Fraser Broth Listeria enrichment broth (Merck, Germany) and pummeled in a Stomacher for 1 min, followed by incubation for 48 h at 30 °C. One loopful of the culture broth was streaked onto chromogenic *Listeria* agar with selective supplement (Oxoid, Hampshire, UK) and incubated at 37 °C for 48 h. Presumptive colonies were streaked onto horse blood agar and TSA plates and incubated at 35 °C for 48 h. The resulting presumptive *Listeria* colonies were submitted for biochemical screening (oxidase test, catalase test and Gram staining).

### Primers and internal amplification control

All oligonucleotide primers used in this study were synthesized by Sigma Company (Singapore). The target genes selected for their characteristic were the variants of *stx1* and *stx2* (Shiga toxin) genes in *E. coli* O157:H7 (Toma et al. 2003)*, inv*A (invasion protein A) gene in *Salmonella* (Rahn et al. 1992) and the *hly*A (transcriptional activator of the virulence factor) gene in *L. monocytogenes* (Shaw et al. 2004), all of which have been reported in recent publications as the most specific and reliable genetic targets for the five pathogens. The sequences of the three primer pairs for the multiplex PCR, their corresponding gene targets and the size of the expected amplification products are also shown in Table 2. In addition, the primer sets targeting the highly conserved regions of the bacterial 16S rRNA gene were employed as an internal control of the presence of amplifiable bacterial DNA.

**Table 2:** Primers selected for the multiplex PCR in this study

Target pathogen	Primer sequence (5′ → 3′)	Gene	PCR product (bp)	Reference
*Escherichia coli* O157:H7	gagcgaaataatttatatgtg	*Stx1, stx2*	518	Toma et al. (2003)
	tgatgatggcaattcagtat			
*Salmonella* spp.	acagtgctcgtttacgacctgaat	*invA*	284	Rahn et al. (1992)
	agacgactggtactgatcgataat			
*L. monocytogenes*	cgcaacaaactgaagcaaagg	*hlyA*	404	Shaw et al. (2004)
	ttggcggcacatttgtcac			
Bacterial DNA	gtattgaaagctctggcgg	16S rRNA	654	Qu et al. (2006)
	tcgcttagtctctgaaccc			

### Template DNA preparation after enrichment

One mL of overnight culture was transferred into a microcentrifuge tube and centrifuged 12,000×g for 3 min and re-suspended by vortex in sterile deionized water. The bacterial suspension was then boiled in 100 °C for 5 min, followed by centrifugation at 12,000×*g* for 2 min. Aliquots of the sample were prepared, frozen at −20 °C and used as a reference template for single and multiplex PCR.

### Optimization of multiplex PCR conditions

The multiplex PCR essay was optimized by varying single parameters while other parameters were maintained. The parameters that were examined were annealing temperature, final primer concentration for each target pathogen, extension time, cycle quantity and Mg^2+^ and dNTP concentrations. Ater PCR, the amplified multiple DNA fragments were separated in a 2 % agarose gel stained with ethidium bromide, electrophoresed, and the DNA was visualized.

### Specificity and sensitivity of the multiplex PCR

The multiplex PCR specificity was checked by examining the ability of the test to detect and distinguish non-target bacterial strains (Table 1) among these three target pathogens. In addition, to archive the method sensitivity, reference strains of *E. coli* O157:H7 (HCMUS), *S. enteritidis* ATCC13076 and *L. monocytogenes* ATCC19111 were grown on LB agar overnight. Small inocula were grown in LB broth for 10 h at 37 °C with shaking, followed by serial dilutions in saline peptone water (Merck, Germany). Each dilution was spread on LB agar to determine the bacterial counts and also prepare DNA by the boiling method as described. The DNA mixture template was made by mixing the five DNA templates with equal concentrations, decimally diluted with each bacterium representing 10^5^, 10^4^, 10^3^, 10^2^, 10^1^ and 1 colony-forming unit (CFU) and subjected to multiplex PCR.

### Examination of artificially contaminated samples

Raw meat pork, chicken and vegetable salads were purchased from a local store. The sample was divided into different parts (25 g for each) and sterilized with radiation. The absence of the five target pathogens in the samples was confirmed by the conventional culture method as described above for each of them. The multiplex PCR assay for the detection of *E. coli* O157:H7, *Salmonella*. spp, and *L. monocytogenes* was evaluated with various food samples in which each pathogen was inoculated at three differing levels as shown in Table 3. 25 g of each sample was transferred into a sterile PE bag containing 225 mL of sterile SEB medium with 1 mL of each level of inoculation of the three target pathogens in each concentration. After each 12-, 18- and 24-h incubation times at 37 °C, a 1-mL aliquot was collected in each period of time incubation, and bacterial DNA was extracted from enriched samples and subjected to multiplex PCR.

**Table 3:** Specificity test for the multiplex PCR assay; a minus (−) indicates the absence of a band and a plus (+) indicates the presence of a band

Species	*stx*	*invA*	*hlyA*	16S rRNA
*E. coli* O157:H7 (NIHE)	+	−	−	+
*E. coli* O157:H7 (HCMUS)	+	−	−	+
*E. coli* O157:H7 (NLU)	+	−	−	+
*Salmonella enterica* ATCC13076	−	+	−	+
*L. monocytogenes* ATCC19111	−	−	+	+
*E. coli* ATCC 11775	−	−	−	+
*E. coli* ATCC 25922	−	−	−	+
*E. coli* (Ahmed et al. 2015)	−	−	−	+
*E. coli* (Amagliani et al. 2004)	−	−	−	+
*E. coli* (Aranda et al. 2007)	−	−	−	+
*E. coli* (Cetinkaya et al. 2014)	−	−	−	+
*E. coli* (Coffey et al. 2011)	−	−	−	+
*C. perfringens* ATCC13124	−	−	−	+
*B. cereus* ATCC11778	−	−	−	+
*S. sonnei* ATCC 9290	−	−	−	+
*V. cholera* ATCC17802	−	−	−	+

*ATCC* American Type Culture Collection*, NIHE* National Institute of Hygiene and Epidemiology, *HCMUS HCM* University of Science*, NLU* Nong Lam University

## Results

### Development of the multiplex PCR protocol

**Fig. 1:**
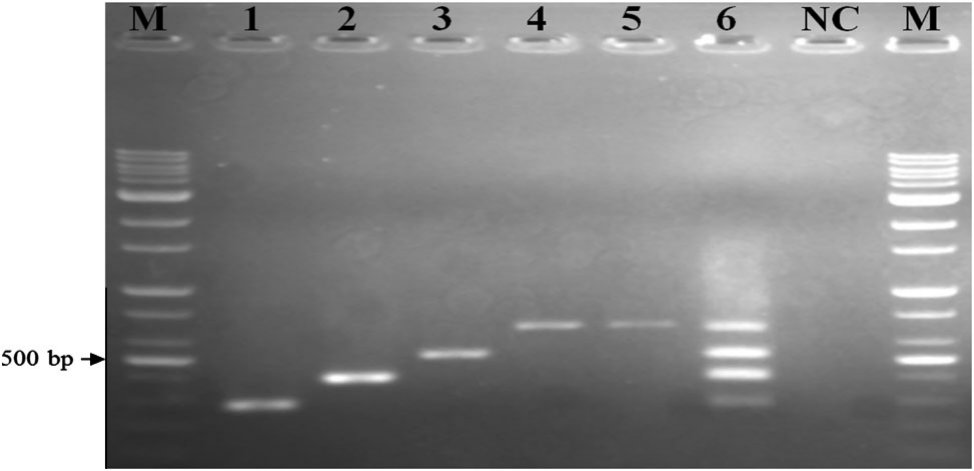
Agarose gel electrophoresis showing monoplex and multiplex PCR-amplified products. M, 100-bp DNA marker;* lane 1*, *Salmonella enteritidis* ATCC13076;* lane 2*, *L. monocytogenes* ATCC19111;* lane 3*, *E. coli* O157:H7 (HCMUS);* lane 4*, 16S rRNA of bacterial DNA;* lane 5*, a mixture of the three DNA templates of pathogens before the optimizing;* lane 6*, the multiplex PCR-optimized conditions with four targets; and NC, negative control

The multiplex PCR products were 284 bp for *Salmonella* spp., 404 bp for *L. monocytogenes*, 518 bp for *E. coli* O157:H7 and an internal control of the presence of 654 bp for 16S rRNA of bacterial DNA, as indicated in Table 2. First of all, the primer pair specificity for each bacterium was analyzed initially by single PCR with the PCR conditions previously published (Qu et al. 2006; Rahn et al. 1992; Shaw et al. 2004; Toma et al. 2003). All species-specific primer pairs produced a single PCR product with an expected product size, indicating species specificity of the used primers (Fig. 1, lanes 1–4). Then, all primer pairs were also tested for simultaneous identification of three of a mixture of the target DNA template of pathogens, using the multiplex PCR conditions based on single performance conditions as follows: 95 °C for 15 min for the initial activation of 12.5 µL of Taq PCR MasterMix (Promega), 0,5 μM of each primer and 20 ng of a mixture DNA template of pathogens in a 50 μL reaction; 30 cycles of denaturing at 94 °C for 30 s, annealing at 58 °C for 90 s, and extension at 72 °C for 60 s; and 72 °C for 10 min for a final extension. The respective bacterial amplicons were produced and separated on agarose gel electrophoresis. Unfortunately, the amplification of multiplex PCR product was not successfully done as faint bands for some amplicons, as shown on lane 5 of Fig. 1. The optimal condition of parameters for multiplex PCR protocol for simultaneous amplification of the three targets, namely, *Salmonella* spp., *L. monocytogenes* and *E. coli* O157:H7, were therefore determined. These parameters of the multiplex PCR assay required careful optimization, including annealing temperature, primer concentrations, extension time, Taq DNA polymerase concentration and Mg^2+^ concentration. The optimum Mg^2+^ concentration for multiplex PCR was examined by adding 1.0, 1.5, 2.0, 2.5 or 3.0 mM Mg^2+^, and finally 2.0 mM of Mg^2+^ was produced for their respective targets. The dNTP concentration was increased stepwise from 100 to 500 mM, and the best results were obtained for 300 mM of each dNTP. 2.0 U was optimal since different concentrations of *Taq* DNA polymerase were tested from 0.5 to 2.5 U in a 50-μL reaction. The concentration of the primer is of critical importance for multiplex PCR; hence, different combinations of primer ratios were subsequently tested, and the primer combination that was optimal for the protocol was 0.15 μM each for *Salmonella* spp., 0.35 μM each for *L. monocytogenes*, 0.25 μM each for *E. coli* O157:H7 and 0.5 μM each for 16S rRNA of bacterial DNA. The optimal annealing temperature was 57.5 °C to amplify simultaneously these four targets in the multiplex mixtures, and a 1.5-min extension with 35 cycles was used to complete the synthesis of all products since adequate resolution of all the amplified products could be seen. The lane 6 of figure 1 shows the yields of the expected multiplex PCR product, corresponding to *Salmonella* spp. (284 bp), *L. monocytogenes* (404 bp), *E. coli* O157:H7 (518 bp), and 16S rRNA of Bacterial DNA (654 bp), that were successfully amplified on agarose gel electrophoresis. Negative control reaction mixtures contained sterile distilled water instead of template DNA. The multiplex PCR-optimized conditions were used in all subsequent experiments.

### Specificity of the multiplex PCR assay

The specificity of the multiplex PCR was conducted with the target strains and non-target bacterial strains. Table 3 shows the amplification products of the expected sizes obtained by PCR on five representative bacterial strains. *E. coli* O157:H7 (HCMUS), *E. coli* O157:H7 (NIHE), *E. coli* O157:H7 (NLU), *S. enteritidis* ATCC13076, and *L. monocytogenes* ATCC19111 that were positive in the multiplex PCR assay, while all non-target strains were negative in the assay and all non-target bacterial including *E. coli* ATCC 11775, *E. coli* ATCC 25922, *E. coli* (Ahmed et al. 2015), *E. coli* (Amagliani et al. 2004), *E. coli* (Aranda et al. 2007), *E. coli* (Cetinkaya et al. 2014), *E. coli* (Coffey et al. 2011), *C. perfringens* ATCC13124, *B. cereus* ATCC11778, *V. cholerae* ATCC17802 and *S. sonnei* ATCC 9290 were negative in the assay, whereas 16S rRNA was amplified as expected. No mispriming or non-specific amplification was observed. Expectedly, the size of each pathogen amplicon was obtained only from the target food-borne pathogens, resulting in 100 % inclusivity and 100 % exclusivity.

### Sensitivity of the multiplex PCR assay

**Fig. 2:**
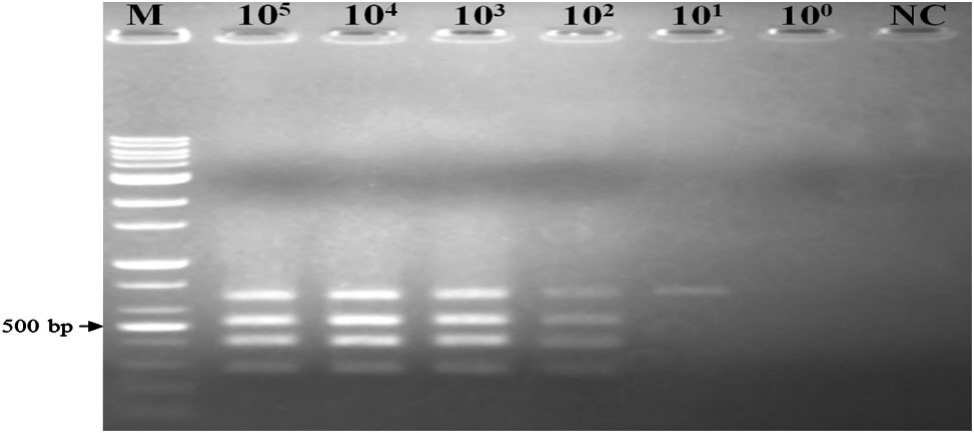
Sensitivity of the multiplex PCR assay on samples obtained from DNA extracts mixed from serial dilutions (10^5^ to 10^0^ CFU/mL) of the three pathogens. *M* 100-bp DNA ladder, *NC*, negative control

The sensitivity evaluation of the PCR assay was carried out using a series of target pathogen genomic DNA in tenfold dilution (from 10^5^ to 10^0^ CFU/mL) of the target pathogens. There was a qualitative decrease in the intensity of the amplicons with the decrease of the DNA concentration. Interestingly, simultaneous specific detection of all three pathogens of all target strains including *E. coli* O157:H7 (HCMUS), *S. enterica* ATCC13076 and *L. monocytogenes* ATCC19111 could be successfully achieved down to 10^2^ CFU/mL (Fig. 2). The multiplex assay developed in this study was effective for the detection of target pathogens.

### Multiplex PCR protocol detection of artificially contaminated samples

**Fig. 3:**
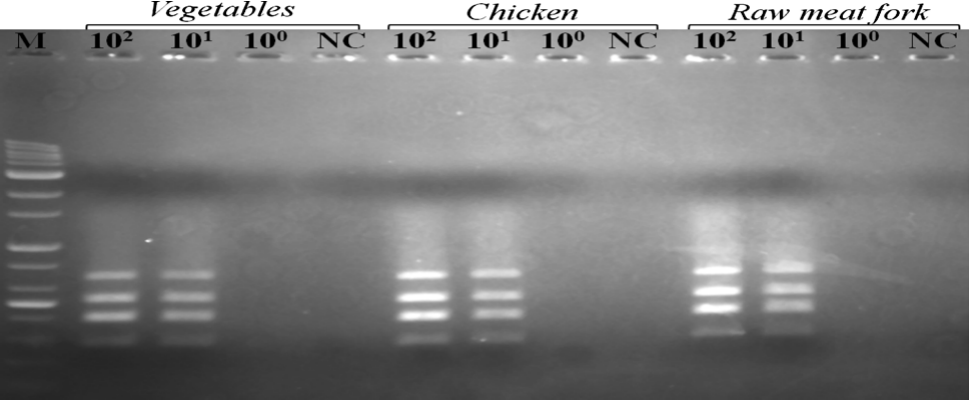
The results of the multiplex PCR assay in the three categories of spiked food samples inoculated with different concentrations of the three pathogen mixtures after 12 h of enrichment. *M* 100 bp DNA ladder, *NC*, negative control

**Fig. 4:**
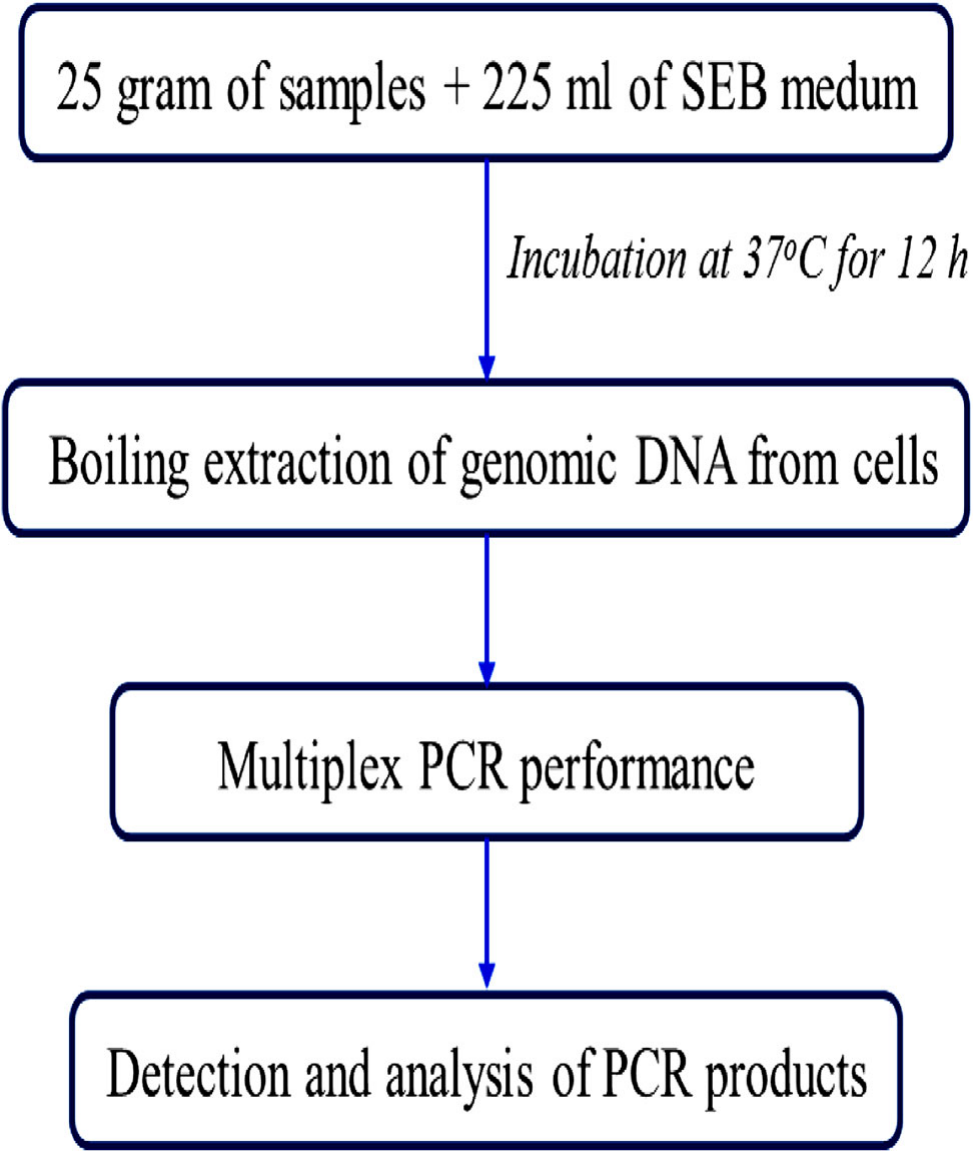
The scheme of multiplex PCR assay for simultaneous detection of *E. coli* O157:H7, *Salmonella* spp., and *L. monocytogenes* in food

To validate and assess the multiplex PCR assay for its application to food samples, the minimum enrichment incubation time and limit of detection of the multiplex PCR using an SEB medium were conducted. Three types of food samples including raw pork, chicken and vegetable salads that were inoculated with *E. coli* O157:H7 (HCMUS), *S. enterica* ATCC13076 and *L. monocytogenes* ATCC19111 with four levels of the number of viable cells (10^0^, 10^1^ and 10^2^ CFU/ml) were analyzed as well as a noninoculated sample as negative control. The results demonstrated that after 12-h enrichment (Fig. 3), the multiplex PCR assay was able to correctly identify the presence of the three food-borne pathogens at all different inoculated levels and down to the lowest concentration of 10^1^ CFU/mL in all three types of food samples as shown in Table 4. Finally, the schematic representation of the steps involved in the multiplex PCR assay for the detection procedure is presented in Fig. 4.

**Table 4:** Multiplex PCR results of three pathogens from artificially inoculated three food sample matrices

Pathogens	Incubation time (h)	CFU/mL	Multiplex PCR results detection in food samples		
			Raw pork	Chicken	Vegetable salads
*E. coli* O157:H7 (HCMUS) (*stx*)	12	0	−	−	−
		10^0^	−	−	−
		10^1^	+	+	+
		10^2^	+	+	+
	18	0	−	−	−
		10^0^	−	−	−
		10^1^	+	+	+
		10^2^	+	+	+
	24	0	−	−	−
		10^0^	−	−	−
		10^1^	+	+	+
		10^2^	+	+	+
*S. enterica* ATCC13076 (*invA)*	12	0	−	−	−
		10^0^	−	−	−
		10^1^	+	+	+
		10^2^	+	+	+
	18	0	−	−	−
		10^0^	−	−	−
		10^1^	+	+	+
		10^2^	+	+	+
	24	0	−	−	−
		10^0^	−	−	−
		10^1^	+	+	+
		10^2^	+	+	+
*L. monocytogenes* ATCC19111 (*hlyA)*	12	0	−	−	−
		10^0^	−	−	−
		10^1^	+	+	+
		10^2^	+	+	+
	18	0	−	−	−
		10^0^	−	−	−
		10^1^	+	+	+
		10^2^	+	+	+
	24	0	−	−	−
		10^0^	−	−	−
		10^1^	+	+	+
		10^2^	+	+	+

## Discussion

One of the 31 common food-borne pathogens identified, whereas bacteria are the primary causes of hospitalizations and deaths in the USA (Scallan et al. 2011). The common food-borne pathogenic bacteria which are responsible for most of the food-borne disease outbreaks are *L. monocytogenes*, *E. coli* O157:H7, *Staphylococcus aureus*, *S. enterica*, *Bacillus cereus*, *Vibrio* spp., *Campylobacter* jejuni, *Clostridium perfringens* and Shiga toxin-producing *E coli* (STEC) (Scallan et al. 2011; Zhao et al. 2014). While the conventional methods used to detect food-borne pathogen are time-consuming and laborious, the methods for rapid detection of food-borne pathogens are becoming more and more important in many food analyses (Díaz-López 2011). One of the most commonly used molecular-based methods for the detection of food-borne bacterial pathogens is PCR. Previously reported methods of multiplex PCR usually assay only for one (two or more target genes) or two pathogens. In the present study, the multiplex PCR for simultaneous identification of *E. coli* O157:H7, *Salmonella* spp. and *L. monocytogenes* strains in food was developed. The lowest detection level such as 10 CFU/mL had been achieved after 12 h of enrichment against three foodborne pathogens since this method was evaluated on three types of samples including raw meat fork, chiken and vegetable. Hence, this multiplex PCR assay combined with a pre-enrichment procedure could reliably and effectively detect the three major food-borne pathogens.

In a multiplexing assay, more than one target sequence, can be amplified by using multiple primer pairs in a reaction mixture. Almost all of the *E. coli* O157:H7 contains *stx1* or/and *stx2* gene, which is a virulence gene encoding a family of related toxins called Shiga (Stx). Generally, the *stx1* or/and *stx2* gene has been proven to be *E. coli* O157:H7 (Gyles 2007; Orth et al. 2007). Hence, in this study, the identification of *E. coli* O157:H7 serotype using *stx* as a target was carried out. According to Toma et al. (2003), the primer *stx* was designed for the specific identification of *E. coli* O157:H7 which detects both *stx1* or/and *stx2* genes, and this has also been applied in different studies (Aranda et al. 2007; Tobias and Vutukuru 2012; Toma et al. 2003). The *inv*A gene of *S. enterica* selected in the study has already been proven to be an important diagnostic tool for detection, as it contains a sequence unique to this genus (Rahn et al. 1992; Shanmugasamy et al. 2011). While the *hly*A gene codes for the action of listeriolysin protein (*hly*A), which was mainly the potential of virulence pathogenic of *L. monocytogenes*, this was considered as the target gene for PCR detection (Dharmendra et al. 2013; Kaur et al. 2007). In addition, all selected primers were tested to examine the possible cross-reactions of primers by homology searches, using the basic local alignment searching tool (BLAST) program, to find the best combination for developing multiplex PCR. Some studies also combined the different primers for all of the targets (Kim et al. 2007; Lee et al. 2014; Rahn et al. 1992; Zhang et al. 2009), considering that they should have similar melting temperatures (*T*_*m*_) and a similar size of target DNA to prevent differential yields in band amplification products. The primer pairs selected in this study were able to allow the successful multiplex PCR amplification after the optimization efforts (Fig. 1).

For specificity testing, the results showed that these four primer pairs in the multiplex PCR assay worked well independently, since multiplex PCR was performed on the mix of DNA extracts from the three food-borne pathogens and was compared to the same test performed on the pathogens individually (Fig. 1). For the optimization of the conditions, based on the yield of PCR products for the four target genes, the results showed that an optimal multiplex was well obtained, as decribed. In addition, the results of the specificity of primers in this study, as shown in Table 3, showed that these four primer pairs were specific for their corresponding target food-borne pathogens and the non-target pathogenic bacteria also did not generate any amplification products. The sensitivity of the multiplex PCR assay was also determined using serial dilutions (from 10^5^ to 10^1^ CFU/mL) of the target pathogens. Interestingly, all target pathogenic bateria could be simultaneously detected down to 10^2^ CFU/mL (Fig. 2). This is more sensitive in comparison with the level of sensitivity of multiplex PCR assay for *E. coli* O157:H7, *Salmonella* and *L. monocytogenes* grown overnight in TSB which was 10^6^ CFU/mL, as shown by Germini et al. (2009), and 10^5^ CFU/mL for *E. coli* O157:H7, *Salmonella*, *S. aureus*, *L. monocytogenes* and *V. parahaemolyticus* grown in Luria–Bertani (LB) by Kim et al. (2007).

In order to assess the detection sensitivity of the multiplex PCR assay for its application to food samples, three type of food samples including vegetable, chicken and raw meat pork were inoculated with different concentrations of three foodborne pathogens and incubated for 12, 18, and 24 h in SEB enrichment medium. The results (Table 4) showed that after 12 h of enrichment, the multiplex PCR assay was able to correctly identify the presence of the three food-borne pathogens at all different inoculated levels and down to the lowest concentration of 10^1^ CFU/mL in these three types of food samples. Comparatively, the highest sensitivity of 10^0^ CFU/mL for four pathogens, such as *E. coli* O157:H7, *L. monocytogenes, S. enterica* and *S. Aureus*, from eight artificially inoculated food samples was obtained after 24 h of enrichment (Zhang et al. 2009). Similarly, the simultaneous detection at concentrations of 10^1^ CFU/mL of five food-borne pathogens including *E. coli* O157:H7, *L. monocytogenes*, *S. enterica*, *V. parahaemolyticus* and *S. aureus* was obtained in the previous multiplex PCR assays (Kim et al. 2007). The multiplex PCR assay described here can simultaneously detect three food-borne pathogens of *E. coli* O157:H7, *Salmonella* spp. and *L. monocytogenes* and was sufficient in specifically detecting 10^1^ CFU/mL in artificially inoculated food samples after enrichment for 12 h.

In conclusion, the multiplex PCR assay was successful in developing a multiplex PCR method that detects simultaneously all of the three major virulence genes from food samples and reliable to detect the presence of *E. coli* O157:H7, *Salmonella* spp. and *L. monocytogenes*. Hence, the multiplex PCR assay has the potential to be used in routine diagnostic laboratories and also as a rapid screening tool in food testing laboratories to quickly identify food samples.
